# Supplementary material for: Functional inactivation of pulmonary MAIT cells following 5-OP-RU treatment of non-human primates
Source: Mucosal Immunol. 2021 Jun 22;14(5):1055–66. doi: 10.1038/s41385-021-00425-3 (PMC8217205; doi:10.1038/s41385-021-00425-3)
Supplement: Supplementary file 1 — Supplementary Information [file 41385_2021_425_MOESM1_ESM.pdf]

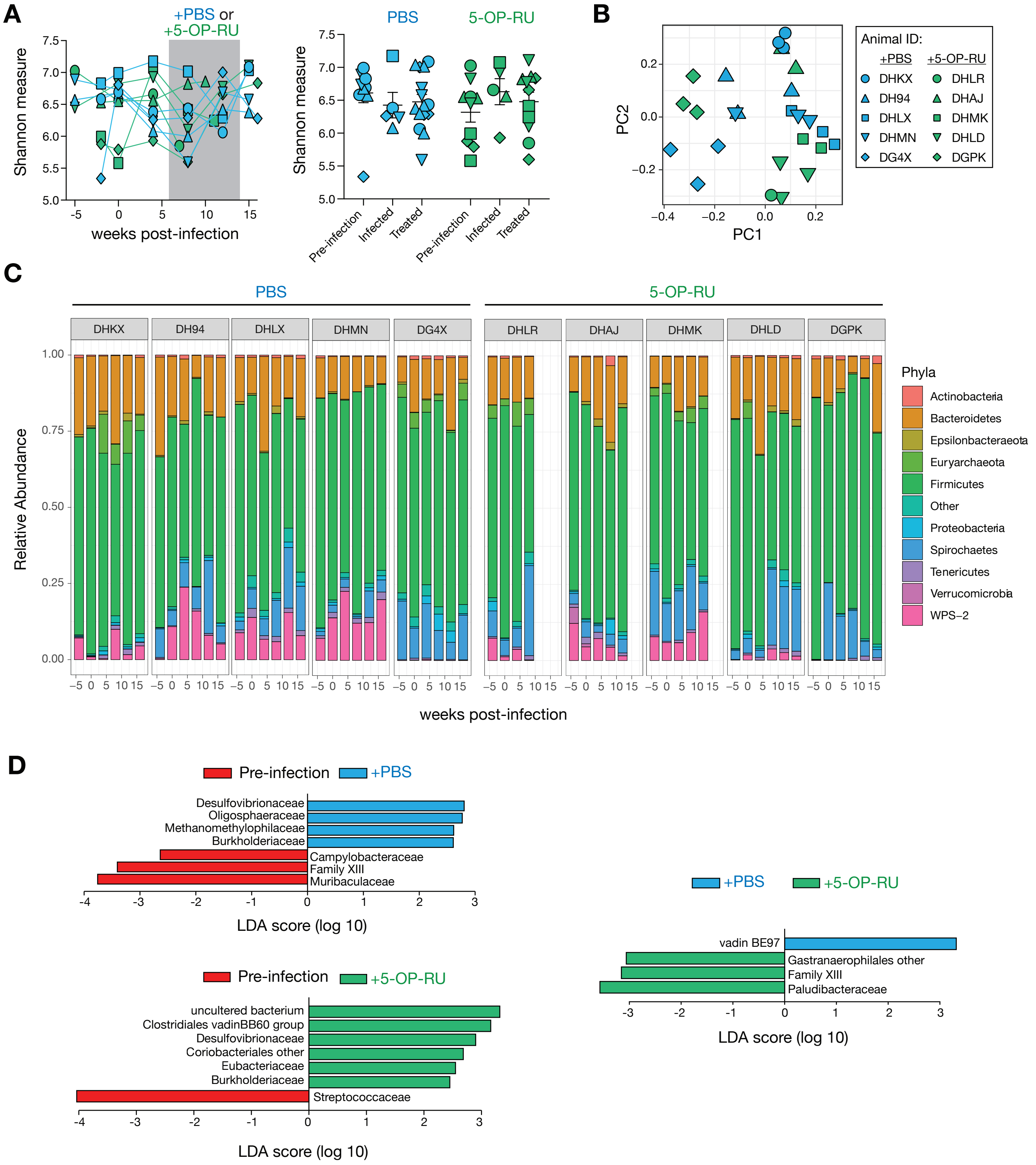

Supplemental Figure 1. 5-OP-RU treatment does not cause major alterations in the intestinal microbiota.
(**A**) Alpha diversity for each sample was estimated using the Shannon index. The left panel represents the diversity of each animal over the experimental timeline and PBS or 5-OP-RU treatment period is highlighted in grey. The right panel shows the pooled pre-infection (week -4/-2, 0), infected (week 4) and treated timepoints (week 8, 12 and necropsy) for the two groups. Statistical testing performed using Student's t-test did not find any significant differences in alpha diversity between the timepoints for either group. Animals and groups can be identified as indicated in the key in B. (**B**) Beta-diversity analysis was performed using the Bray-Curtis dissimilarity matrix on the microbiota composition of the fecal samples collected following PBS or 5-OP-RU treatment. PERMANOVA was utilized to test for statistically significant differences in the microbiomes of the two treatments and was found to be not statistically significant (p-value = 0.055). (**C**) Relative abundance of phyla identified in the fecal samples of each study animal is represented. (**D**) LEfSe analyses depict bacterial families that are differentially abundant between the microbiota of pre-infection and treated timepoints in the PBS and 5-OP-RU groups and between the microbiomes of PBS and 5-OP-RU treated animals ($LDA > 2$, $p < 0.05$).

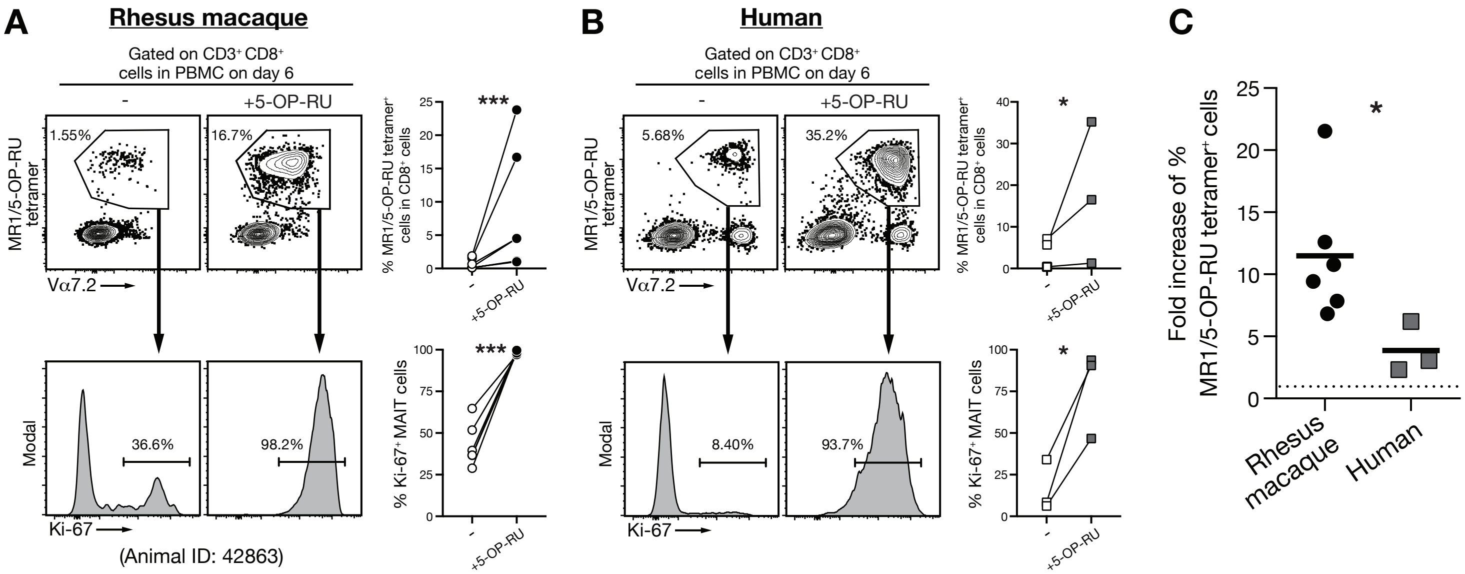

Supplemental Figure 2. Macaque MAIT cells proliferate upon in vitro 5-OP-RU stimulation.
Cryopreserved PBMCs from the uninfected animals (ID: MMX, ZJ29, ZE63, ZG40, H705, 42863) prior to 5-OP-RU treatment or human healthy donors were stimulated with 10 nM 5-OP-RU and cells were harvested on day 6. (**A**) Example FACS plots of MR1/5-OP-RU tetramer staining of cells (*upper panels*) and Ki-67 expressin on MAIT cells (*bottom panels*) in the rhesus macaque PBMCs. Each summary graph was shown on right. (**B**) Example FACS plots of MR1/5-OP-RU tetramer staining of cells (*upper panels*) and Ki-67 expressin on MAIT cells (*bottom panels*) in the human PBMCs. Each summary graph was shown on right. (**C**) Graph displays fold increase in the frequency of MAIT cells in the PBMCs 6 days after stimulation with 5-OP-RU. The fold change in the frequency of MAIT cells was calculated by dividing the frequency of unstimulated cells by the frequency of 5-OP-RU stimulated cells. *$p < 0.05$, ***$p < 0.001$.

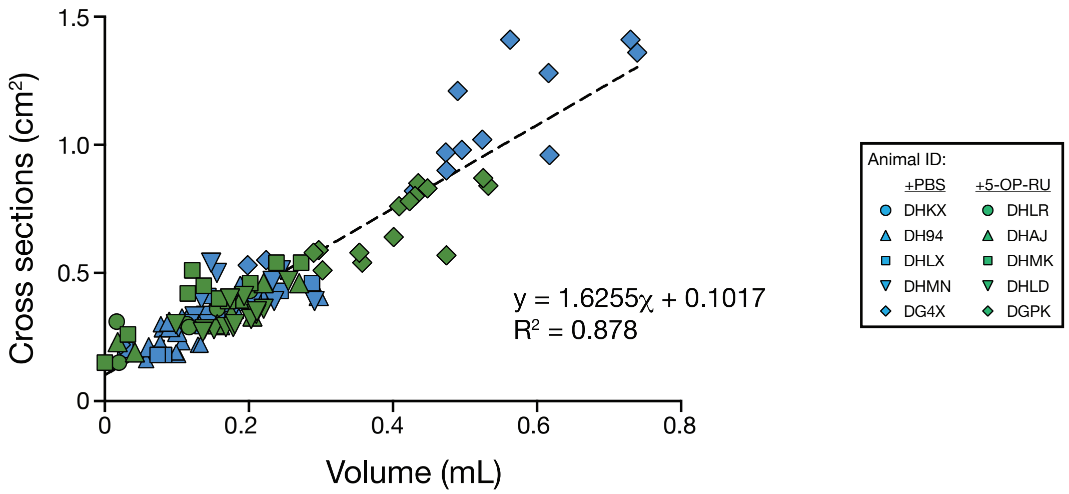

Supplemental Figure 3. Association between the two methods used to estimate bronchial constriction. Cross sections are the result of multiplying the diameter of the bronchus on coronal and sagittal views at a proximal point of the bronchus where constriction usually occurs. Volume was measured from the carinal bifurcation extending linearly 2.3 centimeters along the bronchus. This is the volume of the bronchus directly constricted by the enlargement of the peri-carinal lymph nodes.

Supplemental Table 1. List of flow cytometry panels and antibodies used in this study.

	Antibody/Dye	Clone	Fluorochrome
**Panel 1**	CD8	SK1	BUV395
	CD69	FN50	BUV496
	CD3	SP34-2	BUV805
	Ki-67	B56	BV421
	CD4	SK3	BV510
	Vα7.2	3C10	BV711
	PD-1	EH12	PE
	γδTCR	B1	PE-Dazzle
	CD161	HP3G10	Pe/Cy7
	MR1/5OP-RU tetramer	N/A	APC
	Live/Dead dye	N/A	Fixable Viability Dye 780
**Panel 2**	IFN-γ	Mab11	BUV395
	CD4	SK3	BUV737
	CD3	SP34-2	BUV805
	GM-CSF	BVD2-21C11	BV421
	CD8 (ex)	SK1	BV510
	Vα7.2	3C10	BV711
	TNF-α	Mab11	FITC
	IL-17A	eBIO64DEC17	PE
	γδTCR	B1	PE-Dazzle
	CD161	HP3G10	Pe/Cy7
	MR1/5OP-RU tetramer	N/A	APC
	Live/Dead dye	N/A	Fixable Viability Dye 780
**Panel 3**	TNF-α	Mab11	BUV395
	CD4	SK3	BUV496
	CD95	DX2	BUV737
	CD3	SP34-2	BUV805
	CD28	CD28.8	BV421
	CD8	SK1	BV510
	IFN-γ	Mab11	BV711
	Foxp3	150D	FITC
	PD-1	EH12	PE
	Live/Dead dye	N/A	Fixable Viability Dye 780
